# Sporadic Parathyroid Adenoma: A Pilot Study of Novel Biomarkers in Females

**DOI:** 10.3390/medicina60071100

**Published:** 2024-07-05

**Authors:** Angeliki Cheva, Angeliki Chorti, Kassiani Boulogeorgou, Anthoula Chatzikyriakidou, Charoula Achilla, Vangelis Bontinis, Alkis Bontinis, Stefanos Milias, Thomas Zarampoukas, Sohail Y. Bakkar, Theodosios Papavramidis

**Affiliations:** 1 Laboratory of Pathology, Faculty of Health Science, Medical School, Aristotle University, 541 24 Thessaloniki, Greece; 2 1st Propaedeutic Department of Surgery, Faculty of Health Science, Medical School, AHEPA University Hospital, Aristotle University, 546 36 Thessaloniki, Greece; 3 Laboratory of Medical Biology—Genetics, Faculty of Health Science, Medical School, Aristotle University, 546 36 Thessaloniki, Greece; 4 Department of Vascular Surgery, Faculty of Health Science, Medical School, AHEPA University Hospital, Aristotle University, 546 36 Thessaloniki, Greece; 5 Minimal Invasive Endocrine Surgery Department, Kyanos Stavros, Euromedica, 546 36 Thessaloniki, Greece; 6 Laboratory of Pathology, Interbalkan Medical Center, 546 26 Thessaloniki, Greece; 7 Endocrine & General Surgery, The Hashemite University, Amman 13133, Jordan

**Keywords:** immunohistochemistry, parathyroid adenoma, ANXA2, MED12, MAPK1, VDR

## Abstract

*Background and Objectives*: Parathyroid adenoma is a distinct cause of primary hyperparathyroidism, with the vast majority being sporadic ones. Proteomic analysis of parathyroid adenomas has proposed a large number of related proteins. The aim of this study is to evaluate the immunohistochemical staining of ANXA2, MED12, MAPK1 and VDR in parathyroid adenoma tissue. *Materials and Methods*: Fifty-one parathyroid adenomas were analyzed for ANXA2, MED12, MAPK1 and VDR expressions. Tissue was extracted from formalin-fixed paraffin-embedded parathyroid adenoma specimens; an immunohistochemical study was applied, and the percentage of allocation and intensity were evaluated. *Results*: ANXA2 stained positively in 60.8% of all cell types, while MED12 had positive staining in 66%. MAPK1 expression was found to be negative in total, although a specific pattern for oxyphil cells was observed, as they stained positive in 17.7%. Finally, VDR staining was positive at 22.8%, based on nuclear staining. *Conclusions*: These immunohistochemical results could be utilized as biomarkers for the diagnosis of sporadic parathyroid adenoma. It is of great importance that a distinct immunophenotype of nodule-forming cells in a positive adenoma could suggest a specific pattern of adenoma development, as in hereditary patterns.

## 1. Introduction

Primary hyperparathyroidism (PHPT) affects approximately 1% of the population and is characterized by hypersecretion of parathyroid hormone (PTH) and serum-ionized calcium. There is a female prevalence, with a ratio between men and women of 3–4:1. There are three distinct subtypes of this disease: the most commonly found single parathyroid adenoma (80–85%), multi-glandular hyperplasia (10–15%) and parathyroid carcinoma (1%) [1,2,3,4]. 

Parathyroid adenomas are almost always (90%) sporadic, with the exception of MEN (Multiple Endocrine Neoplasia) syndrome and the HPT-jaw tumor (hyperparathyroidism- jaw tumor) syndrome. Non-syndromic hereditary forms of PHPT, such as FIHPT (familiar hyperparathyroidism), FHH (familial hypocalciuric hypercalcemia) and NS-HPT (neonatal severe hyperparathyroidism) are other causes of PHPT [5]. 

In the international literature, proteomic analysis of parathyroid adenoma tissues has been reported, in which Western blotting, MALDI/TOF (matrix-assisted laser desorption/ionization), mass spectrometry and immunohistochemistry were applied. A vast number of proteins were found to have altered expression in parathyroid adenomas [6,7,8,9,10,11]. 

Annexin-2 (ANXA2) is a 36-kDa protein with NH2—terminal binding ionized calcium, a C-terminal binding actin-F and phosphoinositol, and a central nucleus that creates stable a-helical disks [12,13]. ANXA2 controls cell development and proliferation as well as cell death by interacting with p53 and Nf-kB. Furthermore, ANXA2 regulates cell division, promoting changes in the cytoskeleton, and has been found in increased quantities in the G1-S phase of the cell cycle. It participates in cytoskeleton stabilization and cell interactive activity, as it promotes cell signaling bound with ionized calcium [13]. ANXA2 plays an important role in distinct disease pathogenesis, such as osteoporosis, osteonecrosis in sickle cell anemia and depression, as well as in carcinogenesis, involved in neovascularization and in the development, migration and metastasis of cancer cells [12,13,14,15,16,17]. 

Mediator complex subunit 12 (MED12) constitutes one part of the complex that initiates DNA transcription by binding RNA polymerase II with specific transcription factors and interacting with specific regulatory proteins [18]. It is located in the X-chromosome and is structured by 2212 amino acids and is separated into four distinct parts from the N- to the C-terminal end: leucine part, leucine–serine part, proline–glutamine–leucine part and Opa part [19]. MED12 has been correlated with multiple syndromes such as FG, Lujan, X-linked Ohdo, Opitz–Kaveggia syndrome, as well as neuropsychiatric disorders and tachyarrythmias [20,21,22]. Furthermore, it has been linked to uterine leiomyomas, breast adenomas and phylloid tumors [23,24,25].

Mitogen-activated protein kinase 1 (MAPK1) constitutes a part of the MAPK/Erk signaling pathway. MAPK1 is a 41kDA protein that is involved in mitosis, cell proliferation and death [26,27,28]. Its role in carcinogenesis through epithelial–mesenchymal transition has been extensively studied, while MAPK1 has evolved in hypertrophic cardiomyopathy, coronary artery disease and schizophrenia [28].

Vitamin D receptor (VDR) is a 427 amino acids protein with an N- and C-terminal and a DNA-binding area [29]. VDR acts as a calcitriol mediator in the nucleus, where it forms a heterodimer with retinoid X receptor. VDR is involved in thyroid follicular, colorectal, salivary gland and adrenal gland adenomas [30,31,32,33]. In parathyroid adenoma, it has already been found that VDR expression is decreased and thus in oxyphil cells [6,9].

The aim of our study is to evaluate the quantitative expression of four gene proteins (ANXA2, MED12, MAPK1 and VDR) by immunohistochemical analysis in tissue samples of parathyroid adenoma, as well as their qualitative characteristics.

## 2. Materials and Methods

### 2.1. Tissue Preparation and Assessment

Fifty-nine patients who underwent surgical excision of a single parathyroid adenoma from 2019 to 2022 were enrolled in this study. The diagnosis of primary hyperparathyroidism caused by sporadic parathyroid adenoma was made by endocrinological investigation prior to surgical treatment [34]. Patients with a family history of parathyroid adenoma or Multiple Endocrine Neoplasia Type II syndrome were excluded from our study. All patients provided their consent for participation in the study, which was approved by the local ethics committee. Serum-ionized calcium, phosphorus and parathormone levels were evaluated preoperatively (on the day of the operation) and on the 1st postoperative day.

Specimens’ formalin-fixed paraffin-embedded (FFPE) blocks were obtained and assessed, and 9 patients were excluded due to insufficient material. From the final total of 50 FFPE blocks, 2 µm sections were taken, stained with Hematoxylin–Eosin (H&E), as well as sections on specific positive charged slides applied for immunohistochemical stains.

### 2.2. Immunohistochemical Staining of ANXA2, MED12, MAPK1 and VDR

Afterward, the immunohistochemical staining of ANXA2, MED12, MAPK1 and VDR proteins in parathyroid adenoma was assessed. These specific proteins were chosen according to their association with the formation of other adenomas, either endocrine or not, and the immunohistochemical results were cross-examined with genotyping of specific polymorphisms of these genes so that their association with sporadic parathyroid adenoma to be certified. The antibodies applied were as follows: (a) ANXA2, monoclonal (clone C-10) (from SANTA CRUZ, USA) in a dilution of 1:1000 (45 min) (microwave pH = 6 for 16 min); (b) MED12, rabbit polyclonal (from ATLAS/SIGMA, Sweden) in dilution 1:20 (120 min) (microwave pH = 9 for 20 min); (c) MAPK1, rabbit polyclonal (from ATLAS/SIGMA, Sweden) at a dilution of 1:50 (60 min) (microwave pH = 6 for 16min); (d) VDR, rabbit polyclonal (from SANTA CRUZ USA) at a 1:50 dilution (60 min) (microwave pH = 9 for 20 min). Immunohistochemistry was performed on 2 µm sections on a DAKO Autostainer Link automated platform (DAKO Agilent, USA) with EnVision Flex DAKO detection system (DAKO Agilent, USA). The sections were incubated at 60℃ for 16–18 h, deparaffinized and dehydrated. Afterward, antigen retrieval and incubation with each monoclonal antibody were applied. The evaluation was performed in an optical microscope with 20× and 40× magnification.

Across every immunohistochemical staining, external controls were incorporated. These controls encompassed two essential components: (a) specimens known to elicit a positive reaction to the targeted proteins and (b) specimens exhibiting a negative reaction. This procedure was undertaken to ensure a robust and reliable assessment of the outcome.

For the ANXA2 antibody, lung parenchyma served as the designated external positive control. Similarly, brain substance cells were utilized as the external positive control for the MAPK1 antibody, and glandular epithelial cells of the colon were employed for the MED12 antibody. Lastly, Hodgkin cells from classical Hodgkin lymphoma of the nodular sclerosis type, along with brain cells, were utilized as external controls for the VDR antibody.

Moreover, within each slide of immunohistochemical staining, the intensity of the positive reaction was juxtaposed against endogenous controls, namely cells inherently expressing the specific proteins under examination. In the case of ANXA2 and MAPK1 antibodies, the vascular endothelium was designated as the internal positive control. Conversely, for the VDR antibody, the internal positive control consisted of a hypopopulation of T-lymphocytes (FOXP3+) and sporadic positive nuclei within the normal parathyroid gland, while the vascular endothelium was deemed the internal negative control. The surrounding normal parathyroid tissue was also evaluated for its immunohistochemical reaction with these antibodies.

Staining was scored as follows:

(A) Allocation. The percentage of each type of parathyroid gland cell involved in the adenoma (over all adenoma cells) that was positive for each staining was assessed and scored as follows: i. 0: 0–5% rate; ii. 1: rate 6–30%; iii. 2: 31–70% rate; iv. 3: rate 71–100%.
(B) Intensity. Graded as negative, mild, moderate, intense compared to the internal control (vessels, adipose tissue, normal parathyroid gland): i. 0: negative; ii. 1: soft; iii. 2: moderate; iv. 3: intense.
(C) Type of staining. Staining was scored as cytoplasmic, membranous or nuclear depending on the part of the cell targeted: i. K: cytoplasmic; ii. M: membranous; iii. P: nuclear.

The assessment was carried out by two independent pathologists, and adenomas were classified as positive for allocation and intensity categories 1–3, i.e., >5% allocation and mild to intense intensity.

### 2.3. Statistical Analysis

SPSS statistical package 22.0 (SPSS Inc., Chicago, IL, USA) was used to test frequencies of immunohistochemical staining. One-way ANOVA and the Kruskal–Wallis test were applied to correlate categorical and numeric variables based on the distribution model. For continuous data, we measured means and standard deviations for normal distribution and medians and variances in case of non-normal distribution. A difference at *p* ≤ 0.05 was considered statistically significant.

## 3. Results

A total number of 50 female patients with a mean age of 54.11 ± 12.46 years old were enrolled in this study. The mean diameter of the adenoma was 1.87 ± 0.73 cm, and the mean weight was 1.12 ± 1.04 gr. Table 1 summarizes patients’ characteristics, and Table 2 displays immunohistochemical results.

Τhe percentage of chief, oxyphil and clear cells in H&E staining were determined: (a) 12 chief cell adenomas; (b) 3 oxyphil cell adenomas; (c) 18 chief and clear cell adenomas; (d) 12 chief and oxyphil cell adenomas; and (e) 5 chief, clear and oxyphil cell adenomas. Then, the total percentage of immunohistochemical staining and the positive result for each cell type were assessed.

### 3.1. ANXA2 Immunohistochemical Staining

**Figure 1:**
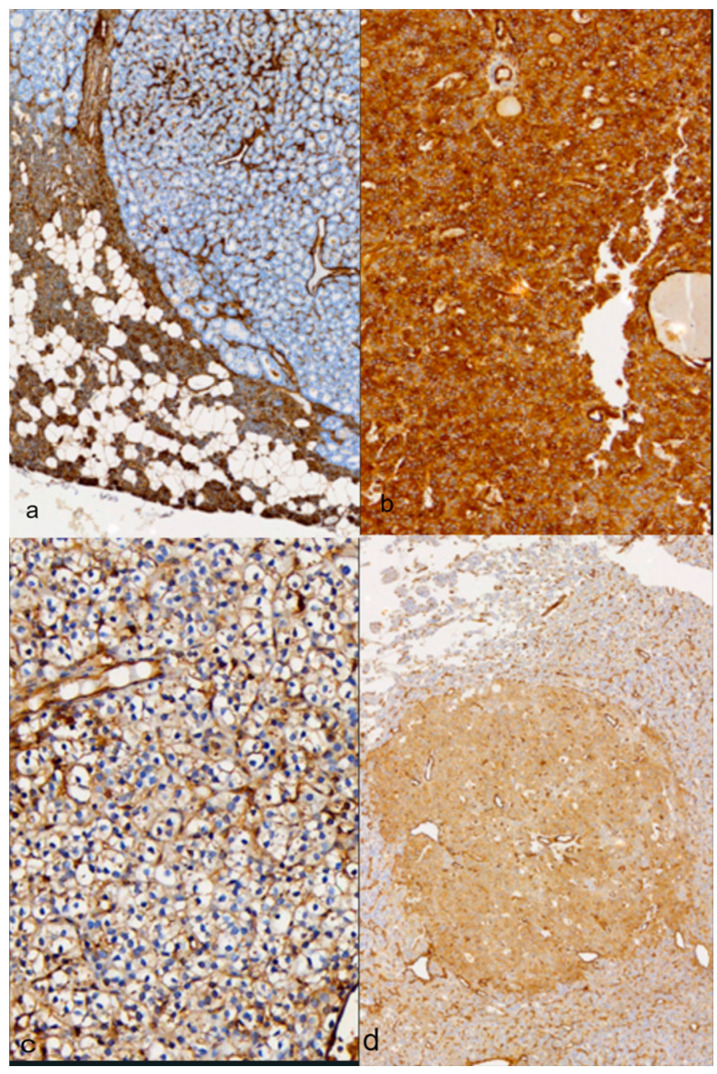
ANXA2 immunohistochemical staining: (**a**) negative staining of chief cells and positive staining of normal parathyroid tissue; (**b**) positive staining of chief cells; (**c**) positive staining of clear cells; (**d**) a distinct immunophenotype of chief cell nodule in an adenoma.

The total percentage of immunohistochemically positive cells with ANXA2 antibody in parathyroid adenoma is 60.8%, which makes ANXA2 positive staining in sporadic parathyroid adenoma (Figure 1).

A total of 51% of chief cells were positive in ANXA2 immunohistochemical staining, with 19.6% showing intense staining, while 46.5% had mild and moderate intensity. The majority of positive chief cells showed mild staining (27.5%). The type of staining in 59.4% was cytoplasmic, 3.9% was membranous, and 21.6% was cytoplasmic and membranous.

For clear cells, the percentage of >5% positive cells was 21.6%, meaning that the majority of cells have negative staining. The intensity of staining is mostly mild, and its type is cytoplasmic.

The majority of oxyphil cells are negative for ANXA staining (62.7%). However, 27.5% of oxyphil cells are positive at a rate of >70%. Staining is mild and cytoplasmic.

In some cases, in the same adenoma, there is a clearly distinct immunophenotype of chief, clear and oxyphil cells.

ANXA2 positive staining was correlated with postoperative serum ionized calcium levels. Thus, when the staining was intense, the postoperative serum ionized calcium levels were higher than in moderate staining (*p* = 0.03).

### 3.2. MED12 Immunohistochemical Staining

**Figure 2:**
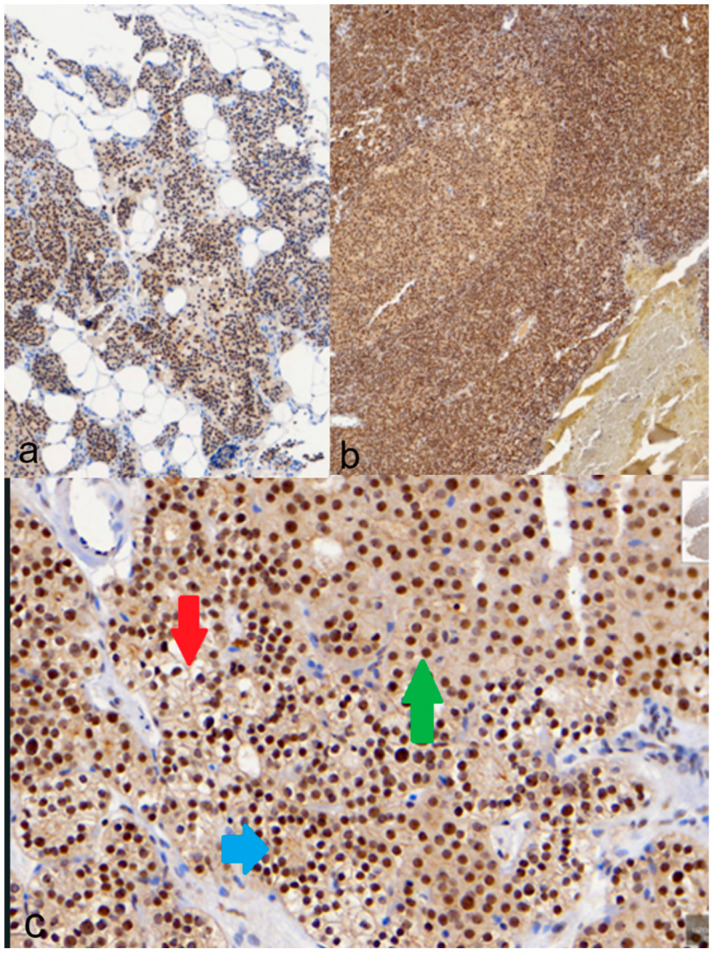
MED12 immunohistochemical staining: (**a**) positivity in normal parathyroid tissue, (**b**) a distinct immunophenotype of oxyphil cell nodule in a positive chief cell adenoma; (**c**) positive chief cells (blue arrow), clear cells (red arrow) and oxyphil cells (green arrow).

Immunohistochemical staining for MED12 is nuclear. The overall rate of positive immunohistochemical staining for MED12 is 66%, demonstrating a positive reaction in the sporadic parathyroid adenoma (Figure 2).

In chief cells, 86.3% have a positive reaction, with 58.8% in the 71–100% positive allocation. The intensity was intense at 27.5%, while a total larger percentage had mild and moderate staining (mild = 23.5%, moderate = 23.5% and mild + moderate = 25.5%).

Regarding clear cells, 33.3% had positive staining, and thus, the positive cells were >70% of the total number of cells. The remaining percentage of cells was negative. The intensity of staining in the majority of cells was moderate at 15.7%.

The majority of oxyphil cells had negative results, with 35.3% of cells having a positive reaction. The intensity of staining was moderate in 19.6%, while mild and intense staining was observed in <10%.

### 3.3. MAPK1 Immunohistochemical Staining

MAPK1 protein seems to follow a different expression pattern as in our study, it was detected with positive expression mainly in the oxyphil cells of the parathyroid gland.

In total, MAPK1 was negative (88.2% of cells are <5% positive).

**Figure 3:**
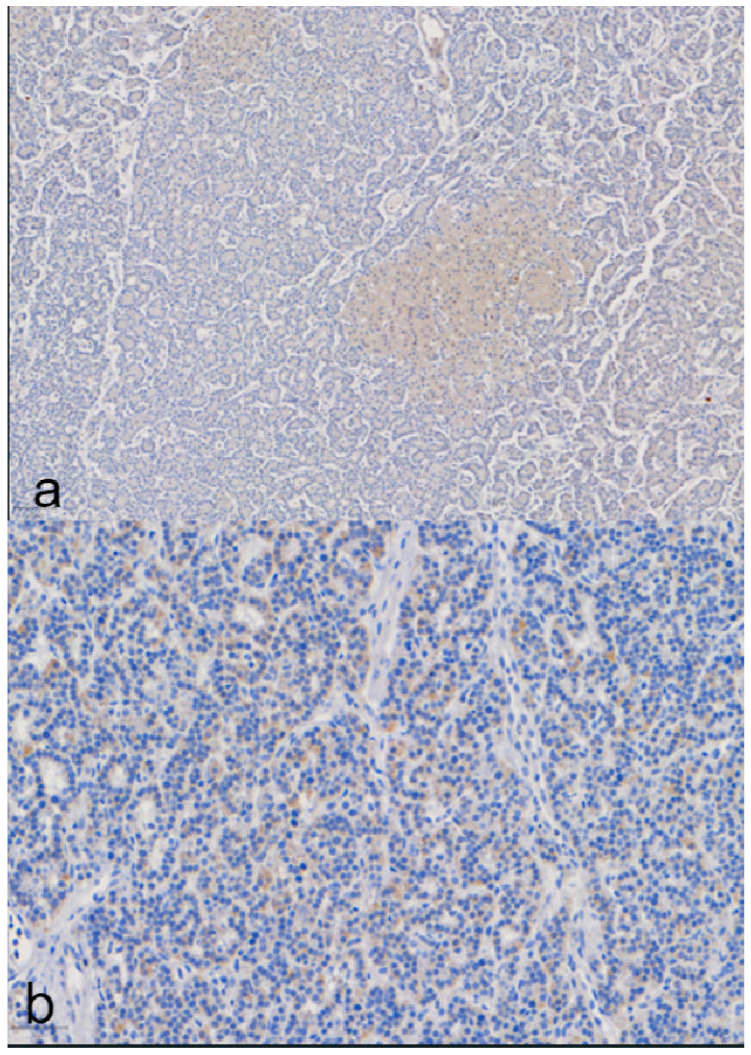
MAPK1 immunohistochemical staining: (**a**) positive oxyphil cells and negative chief cells; (**b**) negative chief cells.

Nevertheless, a trend for oxyphil cells was observed, as oxyphil cells were positive, making it a remarkable observation. Oxyphil cells were positive in 17.7% with mild intensity and cytoplasmic color (Figure 3).

MAPK1 positive staining was correlated with adenoma weight. Thus, in moderate staining, the adenoma was weighted more than in cases of negative staining (*p* = 0.04).

### 3.4. VDR Immunohistochemical Staining

According to the existing literature, VDR staining is nuclear [35,36].

**Figure 4:**
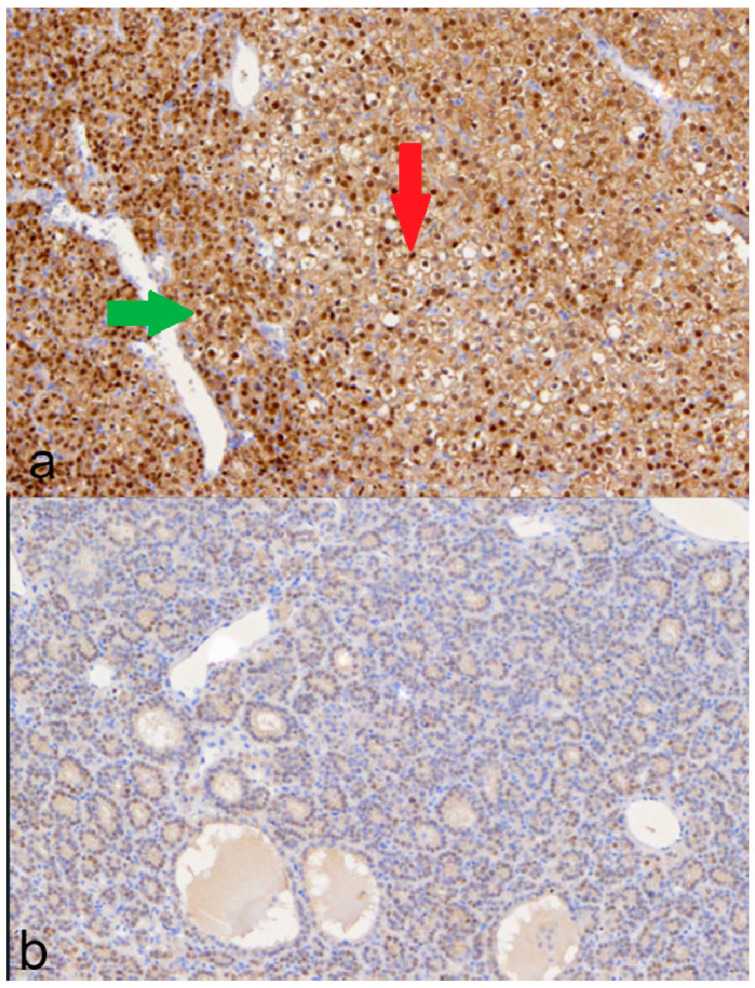
VDR immunohistochemical staining: (**a**) positive nuclear staining in chief (green arrow) and clear (red arrow) cells; (**b**) negative (blue) and positive (brown) nuclear staining in chief cells.

The mean value for positive results based on nuclear staining is 22.8% (Figure 4).

**Figure 5:**
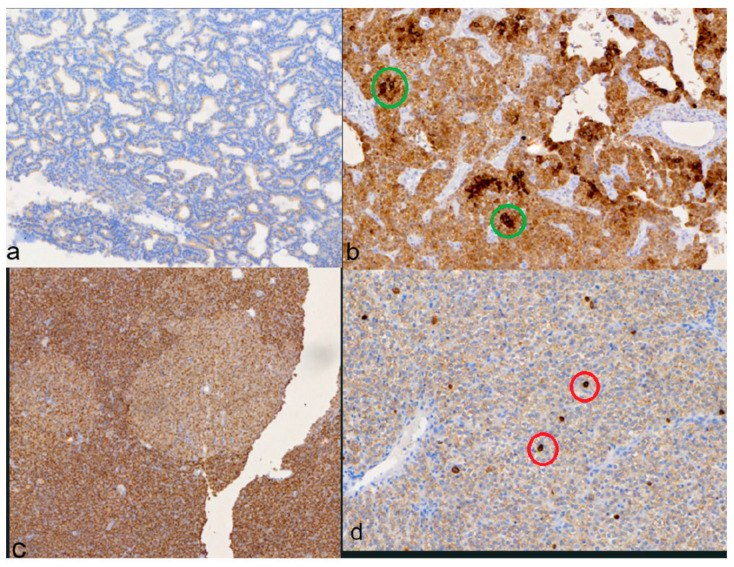
VDR immunohistochemical staining: (**a**) positive membranous staining in the apical side of chief cells; (**b**) positive, intense cytoplasmic staining in group of chief cells (green circle) encircled by positive moderate cytoplasmic staining; (**c**) a distinct immunophenotype of chief cell nodule with mild intensity in an intense positive chief cell adenoma; (**d**) intense positive membranous staining in group of chief cells (red circle) encircled by mild positive membranous staining chief cells.

Finally, a clearly distinct immunophenotype was observed in the same adenoma, with groups of nodule-forming cells being either negative in staining or mild intensity compared to the rest of the adenoma that had an intense reaction (Figure 5).

## 4. Discussion

Immunohistochemical analysis of ANXA2, MED12, MAPK1 and VDR proteins in sporadic parathyroid adenoma confirmed their expression in parathyroid cells. The ANXA2 protein appears to be expressed in all three cell types (main, clear and oxyphil) of parathyroid cells, and immunohistochemical testing revealed a positive staining reaction in adenomas. This result seems to be in line with similar studies in the international literature. Giusti et al. studied the proteins expressed in the normal parathyroid gland compared to the adenoma using mass spectrometry and identified a number of proteins whose expression is altered in the adenoma, including ANXA2, which is triplicated and associated with cell apoptosis [11]. These results were also confirmed by the study by Akpinar et al. with the same methodology, while immunohistochemistry is recommended to confirm the results in these studies [8,10,11].

Furthermore, MAPK1 was found to have weak expression in oxyphil adenomas. The different expression between normal tissue and adenomas was shown to be statistically significant [11]. The MAPK1 signaling pathway seems to play a crucial role in the pathogenesis of parathyroid adenomas and includes a number of other proteins with different expressions in adenomas [7,9,11]. The role of MAPK1 in cell signal transduction seems to be decisive in the pathogenesis of the disease, while the increased expression of mitochondrial proteins in adenomas seems to be related to the conversion of chief cells to oxyphil ones in adenomas and possibly explains the results of our own study [10].

In our study, VDR appeared to be expressed in parathyroid adenoma. The results of the study comparing gene expression in chief and oxyphil cell adenomas by Lu et al. reported reduced expression levels (mild-moderate intensity) of VDR protein in both types of adenomas compared to normal tissue (strong expression) [6]. The reduced intensity of expression of this protein in adenomas was also observed in a previous study by Rao et al. in 2000 [6,37]. This modification is probably related to the increased values of calcium and parathormone in serum (6). In our study, the type of VDR immunohistochemical staining was assessed in two arms: (a) nuclear staining and (b) cytoplasmic and membranous staining. In the international literature, there is a disconcordance of the type of staining for this specific antibody that is considered specific for evaluating the positive reaction in the cells. In the study by An et al., cytoplasmic and nuclear staining is reported in endometrioid carcinoma cells, while in the study by Rehab Mohamed Sharaf et al., cytoplasmic and membrane staining of the VDR antibody in urothelial carcinoma cells is reported [38,39].

The expression of MED12 is reported to be altered in parathyroid gland adenomas in previous studies with mass spectrometry [7]. In our study, MED12 protein expression was found to be positive in the majority of cells, especially in chief cells. Arya et al. reported the expression of this protein in adenoma cells after mass spectrometry [7].

A limitation of our study is the small sample of specimens, as it is formed as a pilot study, and further analysis will be conducted. Another limitation is the inclusion only of women in our study, as primary hyperparathyroidism affects predominantly females. Furthermore, our study does not have a cross-sectional analysis, which could be a possible limitation of our study, but a cross-sectional study is already ongoing.

## 5. Conclusions

ANXA2, MED12, MAPK1 and VDR proved to have a positive staining in the immunohistochemical study of sporadic parathyroid adenomas in varying intensity and allocation percentages. These findings enable the application of these proteins as biomarkers for the diagnosis of parathyroid adenoma. In addition, this study demonstrates that in sporadic parathyroid adenoma, as in hereditary syndromes, there are clearly distinct immunophenotypes of some types of nodule-forming cells in the same adenoma, which probably describes a specific pattern of adenoma development.

## Figures and Tables

**Table 1:** Sporadic adenoma characteristics.

Gender	
Female	50
Age (years)	54.11 ± 12.46
Preoperative serum calcium (mg/dL) (normal values 8.4–10.2)	10.91 ± 0.76
Postoperative serum calcium (mg/dL) (normal values 8.4–10.2)	9.12 ± 0.62
Preoperative parathyroid hormone (pg/mL) (normal values 10–68)	82.77 ± 71.04
Postoperative parathyroid hormone (pg/mL) (normal values 10–68)	18.11 ± 16.99
Preoperative serum phosphorus (mg/dL) (normal values 2.3–4.7)	2.88 ± 0.46
Postoperative serum phosphorus (mg/dL) (normal values 2.3–4.7)	3.38 ± 0.74
Adenoma diameter (cm)	1.87 ± 0.73
Adenoma weight (gr)	1.12 ± 1.04

**Table 2:** Immunohistochemical results (NA = not applicable).

	Total Positivity	Chief Cells			Oxyphil Cells			Clear Cells		
		Positivity	Intensity	Type of Staining	Positivity	Intensity	Type of Staining	Positivity	Intensity	Type of Staining
ANXA2	60.8%	51%	27.5% mild 19.6% intense 46.5% mild and moderate	59.4% cytoplasmic 3.9% membranous 21.6% cytoplasmic and membranous	37.3%	Mild	cytoplasmic	21.6%	Mild	Cytoplasmic
MED12	66%	86.3%	23.5% mild 23.5% moderate 25.5% mild and moderate 27.5% intense	Nuclear	35.3%	19.6% moderate	Nuclear	33.3%	15.7% moderate	Nuclear
MAPK1	11.8%	Negative	Negative	Negative	17.7%	Mild	Cytoplasmic	Negative	Negative	Negative
VDR (all types)	78.5%	NA	NA	All	NA	NA	All	NA	NA	All
VDR (nuclear type)	22.8%	NA	NA	Nuclear	NA	NA	Nuclear	NA	NA	Nuclear

## Data Availability

Data will be available upon reasonable request.
